# Finite Element Modeling and Test of Piezo Disk with Local Ring Electrodes for Micro Displacement

**DOI:** 10.3390/mi13060951

**Published:** 2022-06-16

**Authors:** Yonggang Liu, Shuliang Zhang, Pengfei Yan, Hiji Li

**Affiliations:** 1 School of Mechatronics Engineering, Henan University of Science and Technology, Luoyang 471003, China; yan15713883977@163.com (P.Y.); lihaiji26@163.com (H.L.); 2 Collaborative Innovation Center of Machinery Equipment Advanced Manufacturing of Henan Province, Henan University of Science and Technology, Luoyang 471003, China; sl2226716700@163.com

**Keywords:** piezoelectric disk, finite element modeling, ring electrodes, micro displacement

## Abstract

A new piezoelectric actuator combining interdigitated ring electrodes and a PZT-52(Lead Zirconate Titanate) disk was investigated for the large displacement requirements of piezoelectric actuators. Finite element models were established according to the structural characteristics of the actuator and static analysis was carried out based on ANSYS software. Then Ø25 mm × 2 mm samples were prepared. The displacement detection system was established, and the influence of electrode structure on radial displacement was studied experimentally. A comparison between the experimental results and the finite element analysis confirmed that the finite element model was correct. The results showed that the effect of electrode width on displacement was small. With decrease in electrode center distance and increase in the number of electrodes pairs, the radial displacement increased correspondingly. The peak of radial displacement was 1.63 μm under a 200 V voltage excitation voltage of 0.2 Hz. This was 2.5 times that for a conventional electrode piezo disk with the same structure. The actuator demonstrated better displacement properties. The piezoelectric disk could be valuable in applications involving micro-nano devices.

## 1. Introduction

A piezoelectric actuator is a device that uses the inverse piezoelectric effect of piezoelectric materials. Such actuators can convert electrical energy into mechanical energy under an electric field and produce precision mechanical displacement [1,2,3]. High precision micro-drive and micro-positioning technology are needed in the technical fields of optical engineering [4], microelectronic manufacturing [5], aerospace [6], ultra-precision manufacturing [7], micro-robotics [8,9], medicine and genetic engineering [10,11]. Precision actuators with a piezoelectric component as the core show micro/nano displacement resolution. They play an important role in submicron and micro/nano ultra-precision drive and control technology [12,13,14]. Piezoelectric ceramics possess the advantages of convenient control, high power density, high displacement resolution, fast frequency response, low power consumption, no noise, and strong anti-electromagnetic interference ability [1]. They have developed into a unique driving form in the field of precision drive and are applied in automatic assembly devices, micro-robot operation, micro-machinery manufacturing, precision optical adjustment, precision positioning, nano-machining and optical fiber operation [15,16,17,18].

The displacement of a piezoceramics actuator is relatively small, which limits their application. To increase the displacement, some displacement amplification methods have been proposed. Typical composite structures include thunder [19], rainbow [20], honeycomb [21,22], Moonie-type [23,24], cymbal-type [25,26], active fiber composites [27,28] and micro-fiber composites [29,30,31,32,33]. Using the cumulative effect of multiple piezoelectric sheets, piezoelectric stacks have been widely used in the field of precision micro-drives. However, due to limitations in structure, piezoelectric stacks are not easy to connect into a large mass inertia block, so their driving ability is limited [2,34,35]. To increase the driving capacity, multiple piezoelectric stacks are usually required, and the cost of the driver is high. Moreover, the running speed of piezoelectric stacks is relatively low [1]. For the above reasons, piezoelectric actuators with micro displacement and low driving cost have become a focal area of research [36,37,38,39,40].

Interdigital electrodes are among the most used periodic electrode structures and are used in microelectromechanical systems, telecommunications, chemical sensing, piezo acoustics, and biotechnology. The advantages of interdigital geometry have attracted device designers [41]. Sinkevicius et al. [4] combined piezoelectric ringing with a Pockels cell. The piezoelectric ringing amplitude distribution in the aperture of the Pockels cell was measured. Tu et al. [42] designed flexible interdigital capacitive sensors using a 3D printing and spraying process. The interdigital circuits of the structure were printed with conductive silicone rubber filled with silver-coated glass fiber and carbon fiber. The effect of the spaces between IEs was studied. The response mechanism was modeled based on the evolution of the microstructure of the sensors. Lai et al. [43] presented a multiparameter theoretical model by using a parallel plate capacitance model. The influence of dimensional, structural, and material parameters on the force generated by the electro adhesive pad with interdigitated electrodes was investigated. A 3D simulation model was created in COMSOL software (COMSOL Ltd., Stockholm, Sweden) to verify the accuracy of the above theoretical results. Zhang et al. [44] used 3D interdigital electrodes to improve the performance of aligned nanofiber-based piezoelectric nanogenerators. The way in which the output voltage and current were affected by the nanofibers and electrodes was studied. The performance was optimized by fabricating interdigital electrodes with different densities of microstructures. Tian et al. [45] proposed wave number–spiral acoustic tweezers to achieve programmable particle/cell manipulation. Some functions were demonstrated experimentally including multiconfiguration patterning, parallel merging, pattern translation, transformation, and rotation, as well as the dynamic translation of single microparticles along complex paths.

In this paper, a type of LRE (local ring electrode) piezoelectric disk was studied to generate radial expansions and contractions in a plane using the piezoelectric constant d_33_. Finite element modeling was adopted to analyze the displacements of the piezoelectric rings and the piezo actuator, respectively. Displacement experiments were carried out to study the effect of the electrode structure on the displacement response of the piezoelectric disk.

## 2. Structural Principle of Actuator

**Figure 1:**
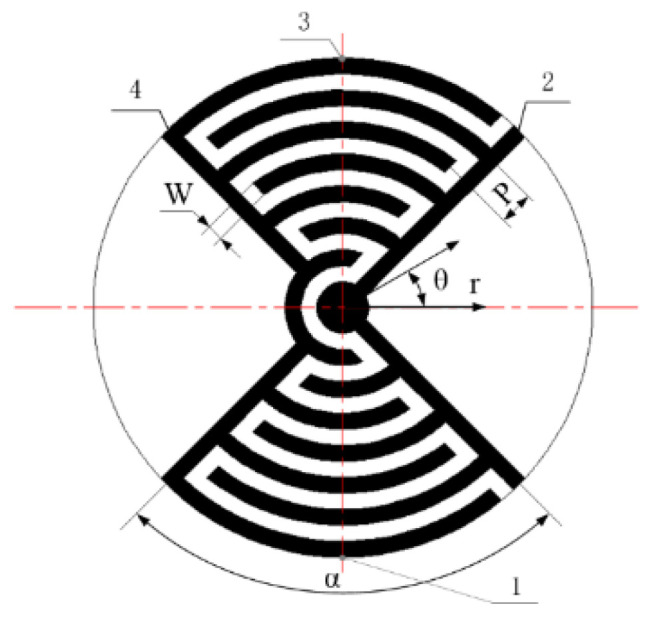
Schematic of an LRE piezo disk: 1. the constraint region; 2. the negative main electrodes; 3. the displacement monitoring mark; 4. the positive main electrodes.

An LRE piezo actuator in the shape of a circular column is shown schematically in Figure 1. The disk surface is divided into an electrode region and a non-electrode region. The interdigitated ring electrodes are concentrated in a certain district so that the driving signal can be output centrally. The electrode consists of a series of branch electrodes drawn from the positive and negative main electrodes, respectively. The electrodes on the upper and lower surfaces are in exactly the same position.

The electrode region is arranged in two opposite regions at 90° to each other, making the deformation more concentrated and directional. A cylindrical coordinate system is adopted consistent with the structural features, where W is the electrode width, P is the center distance of the branch electrodes and α is the electrode region angle. The constraint region is the constraint and location as determined by finite element analysis and displacement experiments. The displacement monitoring mark is the site where the displacement was extracted in finite element analysis post-processing, and is also the detection point identified in an actuator displacement test.

**Figure 2:**
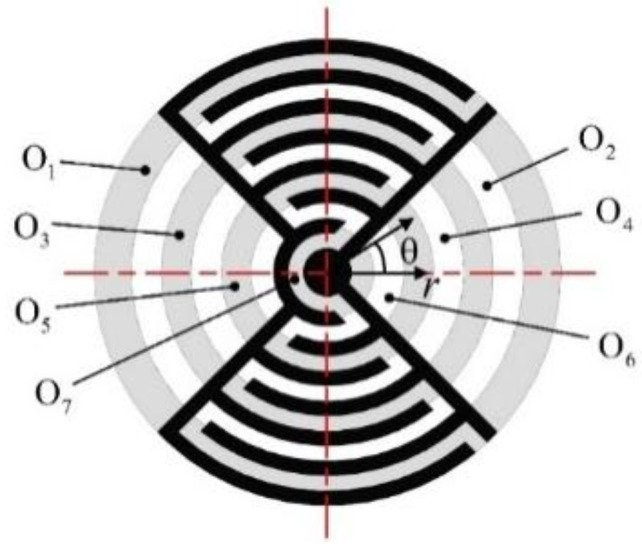
Piezo-ring-pairs.

Due to the characteristics of the electrode, the disk is divided into rings along the centerline of the branch electrodes, as shown in Figure 2. A type 1 piezoelectric ring includes O_1_, O_3_, O_5_ and O_7_. These have similar electric field distributions and the same polarization axis. They are assumed to have the same piezoelectric constants. A type 2 piezoelectric ring includes O_2_, O_4_ and O_6_. These have similar electric field distributions and the same polarization axis. These are assumed to have the same piezoelectric constants. The two piezo-ring pairs have opposite polarization directions. The piezoelectric constants are opposite to each other. Because of the uniformity of the material, it is assumed that the mechanical constants of all piezo rings, including the elastic modulus and the Poisson ratio, are the same.

When the LRE piezo disk was applied to an actuator, the radial displacement and radial force were superimposed by piezo-ring pairs. This structure is mechanically connected in series and electrically connected in parallel.

## 3. Finite Element Analysis

### 3.1. Piezo Ring Analysis

**Figure 3:**
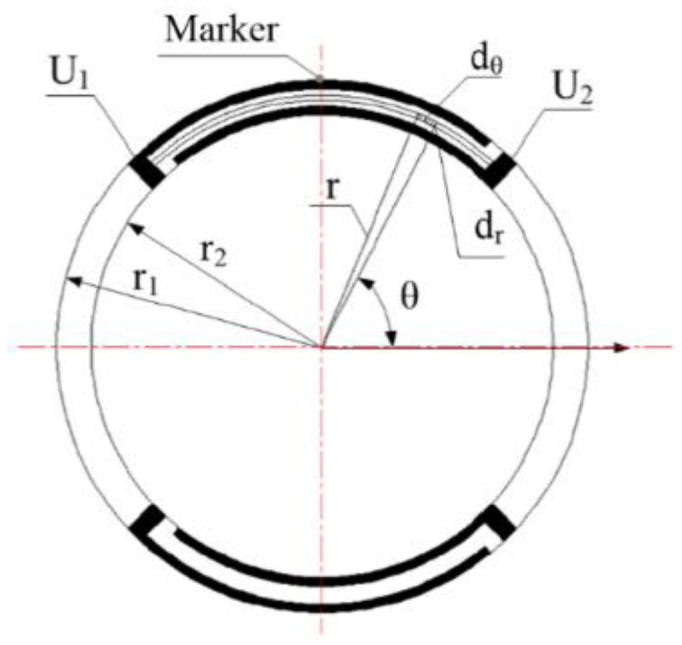
The characteristic piezo ring.

According to the structural characteristics of the disk, a characteristic piezo ring was selected for study, as shown in Figure 3.

There is a reduced electric field in the electrodeless region. Therefore, this region cannot be polarized. The electric field in the electrode area is relatively large. The electrode area can easily be polarized. The remanent polarization intensity is large. The piezoelectric constant and dielectric constant are related to the polarization strength. To simplify the analysis, it was assumed that the electrode region of the disk was fully polarized and had the same piezoelectric constants and dielectric constants. PZT-52 was adopted as the base piezo disk. The dielectric constant and piezoelectric constant of PZT-52 observed are shown in Table 1.

A piezo disk of Ø25 mm × 2 mm in size was used. For the electrode structure, α was 90°, W was 0.8 mm and P was 1.6 mm. Seven electrode rings were arranged on the surface of the element. The outer radii r_1_ of the piezo rings O_1_~O_7_ are shown in Table 2.

**Figure 4:**
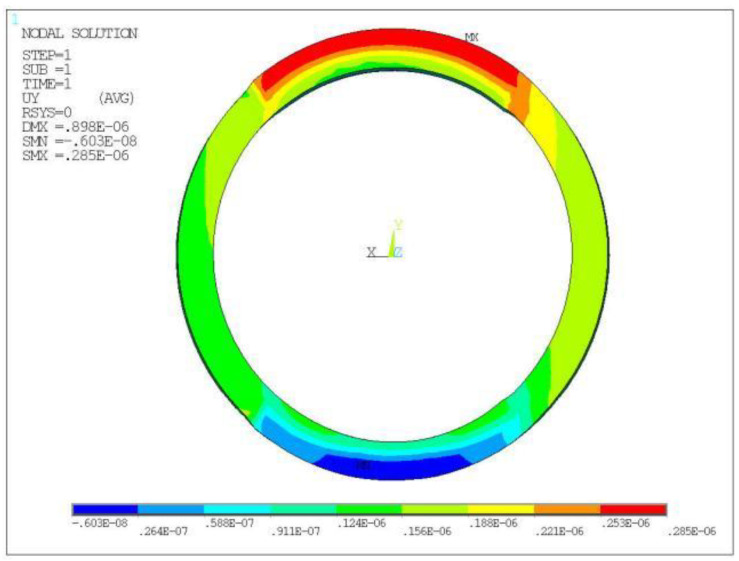
The displacement (m) cloud of piezo ring O_1_.

To study the effect of the radius on the displacement of the piezo ring, a finite element model of the piezo ring O_1_ was established. A 10° arc surface near 270° of the piezo rings, as shown in Figure 3, was selected as the positioning surface. It was assumed that the piezo ring was not affected by the others. The piezo ring was analyzed statically with ANSYS (ANSYS Inc., Pittsburgh, PA, USA). A static voltage of 200 V was applied to the positive electrode and 0 V to the negative electrode of the piezo ring. The displacement cloud of O_1_ obtained is shown in Figure 4. The maximum displacement was 0.29 μm near 90° of ring O_1_. The radial displacement of the ring was not uniform due to the large and non-uniform mesh.

**Figure 5:**
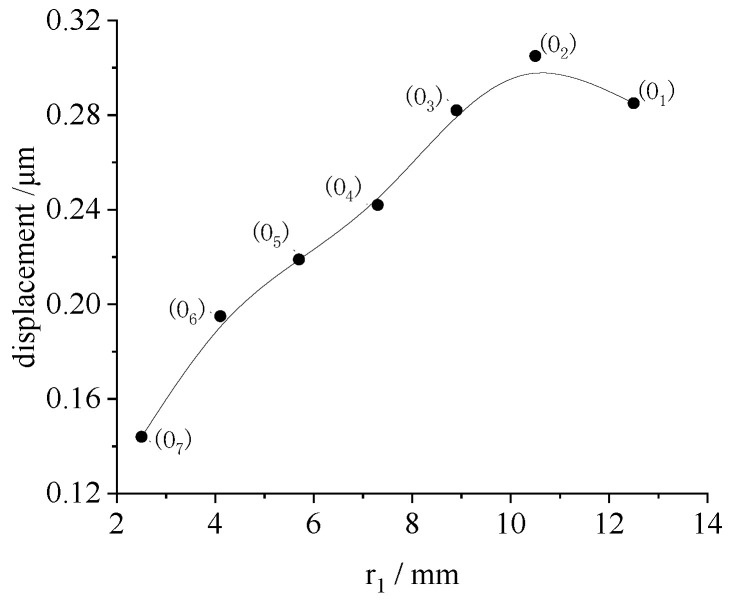
The maximum displacements of piezo ring versus the radius.

To study the effect of the radius on the displacement of the piezo ring, the displacement of rings O_1_~O_7_ were also analyzed statically with ANSYS. The maximum displacements under 200 V voltage are shown in Figure 5. Under the same loading and constraint conditions, the displacement of the piezo ring of the outer circle was large. The peak displacement increased linearly with increase in the radius. When the radius exceeded 10.5 mm, the peak displacement was stable.

### 3.2. Single Electrode Ring Loading Analysis

**Figure 6:**
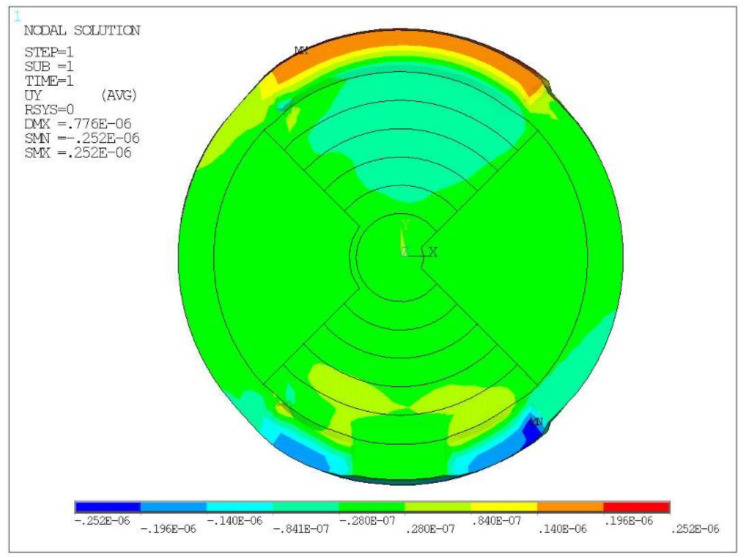
The radial displacement (m) of disk with O_1_ loading.

According to the structural characteristics of the LREs piezo disk, an overall model was established in ANSYS. The 10° arc surface near 270° of the LREs piezo disk, as shown in Figure 1, was constrained. To analyze the influence of single ring loading on the displacement of the actuator, a single electric ring loading analysis was carried out. A voltage of 200 V was then applied to the positive electrode of the piezo ring, and 0 V was applied to the negative electrode. A static analysis was performed to obtain the radial displacement cloud of the LRE piezo disk produced by a piezo ring, as shown in Figure 6.

It can be seen from Figure 6 that the displacement of the restricted region at the lower end of the LREs piezo disk was essentially zero. The piezo ring O_1_ produced a maximum radial tensile displacement of 0.25 μm. The piezo rings O_2_~O_7_ were not excited, so the radial displacement was small. A similar static analysis of the other piezo rings was performed separately. The algebraic sum of the individual displacement peaks was 1.47 μm when W was 0.8 mm, P was 1.6 mm and the electrode ring number was seven.

### 3.3. Full Electrode Loading Analysis

**Figure 7:**
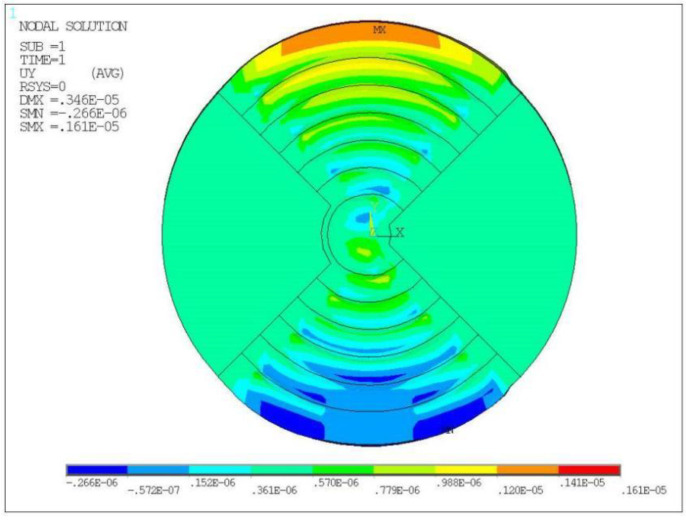
Radial displacement (m) of disk under full electrode loading.

The LRE piezo disk was loaded with full electrodes, and each piezo ring was simultaneously excited by a voltage to study the radial displacement. The positive electrodes of all piezo rings were loaded with 200 V, and the negative electrodes were loaded with 0 V. The constrained area was unchanged, and a static analysis was performed. The displacement is shown in Figure 7. Due to the accumulation of piezo ring displacements, the maximum displacement of the disk reached 1.61 μm at the displacement monitoring mark. The displacement values of the full-electrode excitation were greater than the sum of the displacements of the single-electrode rings excitation separately. This showed that the displacement of the disk was not an algebraic sum of the displacements of each ring.

## 4. Displacement Test and Results Analysis

### 4.1. Experimental Device

**Figure 8:**
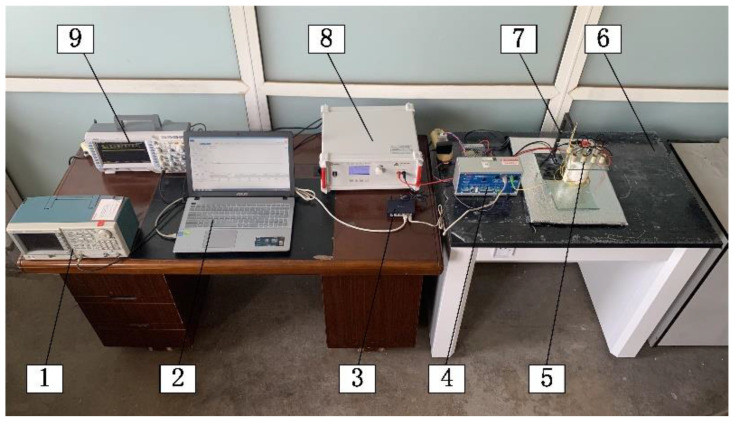
Experimental platform for actuator displacement detection: 1. Tektronix AFG1022 signal generator (Tektronix, Inc., Johnston, USA); 2. Computer; 3. Pulian TP-LINK switch (TP-Link Ltd., Shen, China); 4. Spectral confocal controller IFC2451 (Micro-Epsilon Measurement Technology Management Company Group. Bavaria, Germany); 5. Samples; 6. Three-stage shock-absorbing stand; 7. Laser probe IFC2403-0.4 (Micro-Epsilon Measurement Technology Management Company Group. Bavaria, Germany); 8. Antai ATA-2021 high voltage amplifier (Aigtek Electronic Technology Ltd., Xian, China); 9. Tektronix TDS2024C oscilloscope (Tektronix, Inc., Johnston, IA, USA).

PZT-52 powder was selected. Samples of Ø25 mm × 2 mm were prepared using an atmospheric pressure-sintering process. Electrodes were fabricated by screen printing. Thermal polarization was adopted to polarize the samples. To detect the displacements of the samples, a displacement detection experimental platform was built, as shown in Figure 8. It included an excitation signal device, a displacement acquisition and processing device, a computer for signal recording and analysis, and a shock absorption stand.

**Figure 9:**
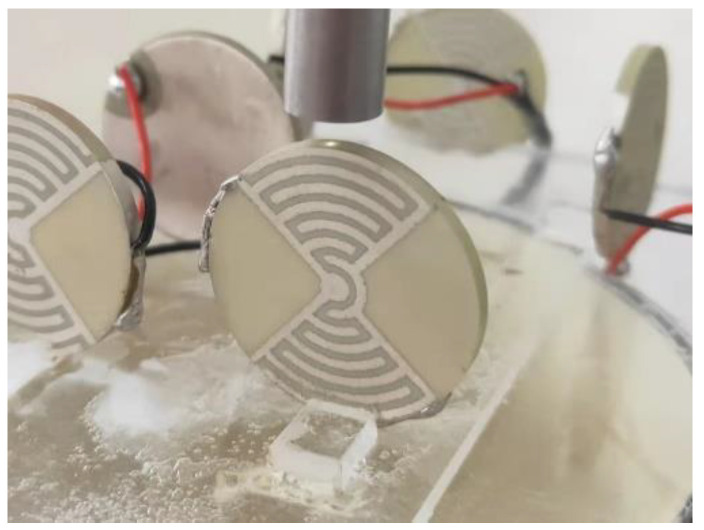
Installation position of the sample and the laser head.

One end of the sample was fixed on an acrylic sheet, and the other end was free. The samples were put together on the shock-absorbing stand. The laser probe was fixed on the bracket. The light source was focused on the displacement monitoring mark at the top of the sample. When the radial displacements were detected, the installation position of the sample and the laser head were as shown in Figure 9. The laser head was 2 mm away from the mark. During the test, the excitation signals were amplified by the high-voltage amplifier. The signals were connected to the oscilloscope at one end. The other end was connected to the positive and negative poles of the samples to be tested. A switch was connected to the spectral confocal controller and the computer at the same frequency to collect the displacement data. The probe and samples were placed separately from the other devices. They were placed individually on the stand. This was to reduce the influence of noise on the experimental data.

### 4.2. Result Analysis

#### 4.2.1. Displacement Response of Single Electrode Ring Excitation

The linear range of the probe was 400 μm. The starting point of the measuring range was 2.5 mm. The measurement error was 0.3 μm and the resolution was 16 nm. This enabled assessment of the radial displacement of the sample. The positive branch electrodes of the LRE piezo disk were connected as a whole, as were the negative branch electrodes. It was necessary to first cut off other branch electrodes when the displacement of the LRE piezo disk driven by a piezo ring was detected. In this way, only the positive and negative electrodes of the given piezo ring could be energized.

**Figure 10:**
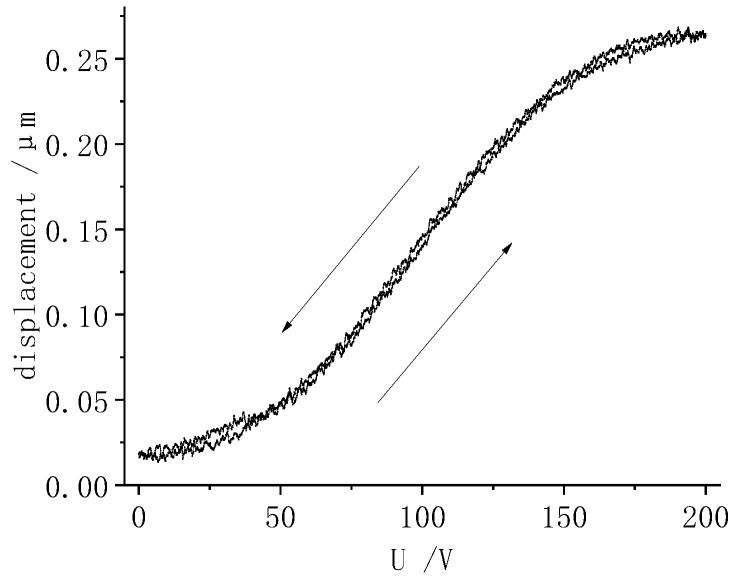
Displacement response when the O_1_ is loaded.

A −200~200 V sinusoidal voltage with a frequency of 0.2 Hz was applied to a given piezo ring. The displacement response curve of the sample was obtained, as shown in Figure 10. As can be seen from Figure 10, the displacement curve of the middle section is nearly linear. The radial tensile displacement peak was 0.27 μm.

**Figure 11:**
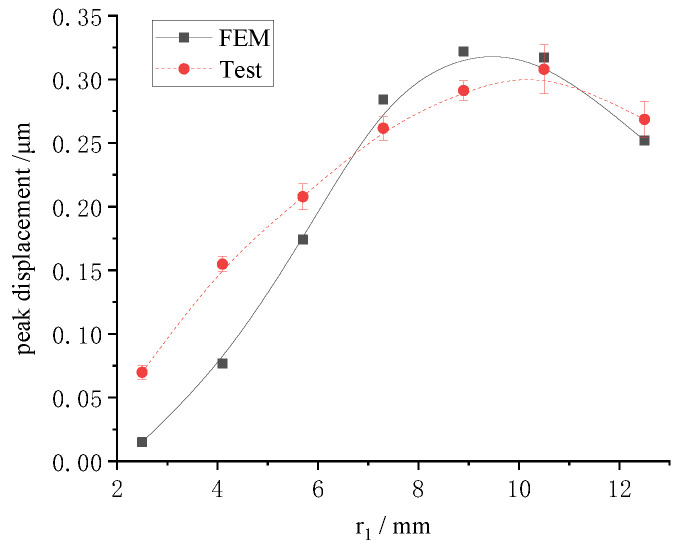
The radius effect on the peak displacement of disk when each piezo ring is excited individually.

To study the displacements of the LRE piezo disk, the piezo rings O_1_~O_7_ were energized separately. The radial displacements of the sample under single-electrode-ring excitation were detected. The effect of the piezo ring radius on the peak displacement is shown in Figure 11. For comparison, the displacement results of the single-electrode-ring loading in Section 3.2 are summarized in Figure 11.

The law of displacement for the radius obtained in the finite element analysis and the experiment was basically the same. This showed that the radial displacement of the disk was not a linear function of the radius. As the outer radius of the piezo ring increased, the radial displacement of the disk gradually increased from the inside to the outside. The algebraic sum of the displacement peak, when all electrode rings were excited individually, was 1.56 μm.

#### 4.2.2. Micro Displacement Analysis

**Figure 12:**
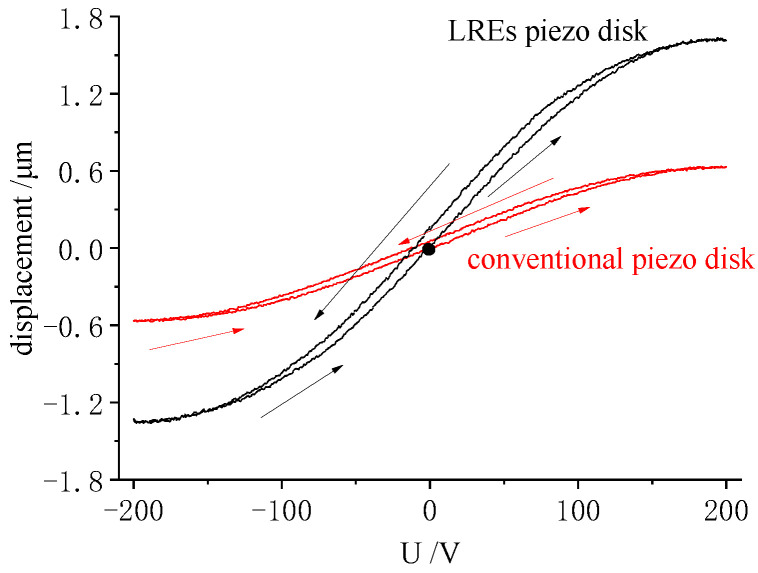
Displacement response of LRE piezo disk and conventional piezo disk.

According to the results of the displacement test, LRE piezo actuators were prepared, with parameters for the electrode as follows: α—90°, W—0.8 mm, P—1.6 mm, and an electrode pair value of seven. For comparison, conventional piezo actuators of the same size were prepared, with fully covered electrodes on the upper and lower surfaces. The samples were loaded with a sine of 0.2 Hz, −200~200 V; the displacement response is shown in Figure 12.

In Figure 12, the peak value of radial tensile displacement of the LRE piezo actuator was 1.63 μm. The value was larger than the 1.56 μm for the sum of the peak displacements in the test of single electrode ring excitation described in Section 4.2.1. This showed that the radial displacement response of the disk was not a linear superposition of the ring displacements. When the full electrodes were loaded, the piezo rings squeezed and stretched each other.

The radial tensile displacement peak of the conventional electrode piezo actuator was 0.64 μm. The displacement of the LREs piezo actuator was 2.5 times that of the conventional electrode piezo disk. It was evident that the LRE piezo actuator exhibited significant displacements. Since the displacements of the LRE piezo actuator were superimposed on the displacements of the individual piezo rings, they exhibited more obvious hysteresis characteristics. There was a clear gap between the ascending and descending segments of the displacement curve. The rising and falling segments of the displacement response curve of a conventional actuator essentially coincided.

#### 4.2.3. Effect of Electrode Width on Displacement

**Figure 13:**
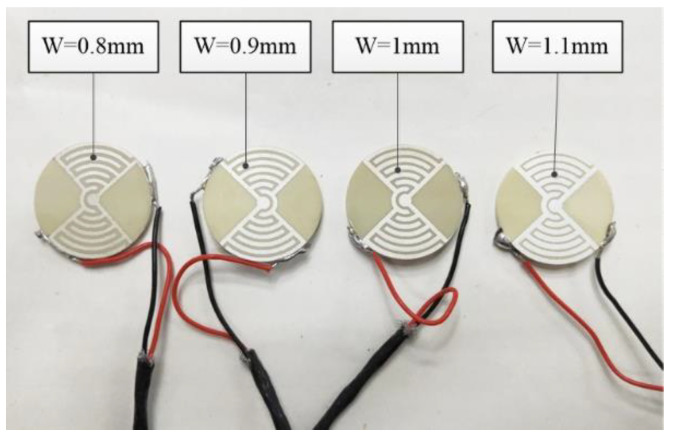
LRE piezo sample with different electrode widths.

The electrode width is a key dimension. It determines the electric field strength of the sample. In the process of sample preparation, any errors in the width will impact the polarization of the piezoelectric materials. This will affect the piezoelectric performance of the sample. Therefore, the effect of electrode width on the displacements of LREs piezoceramic disk needs to be studied. Some LRE piezo samples were prepared. The electrode widths were 0.8 mm, 0.9 mm,1 mm and 1.1 mm. The electrode center distance was 1.6 mm. There were seven pairs of electrodes. The samples are shown in Figure 13.

**Figure 14:**
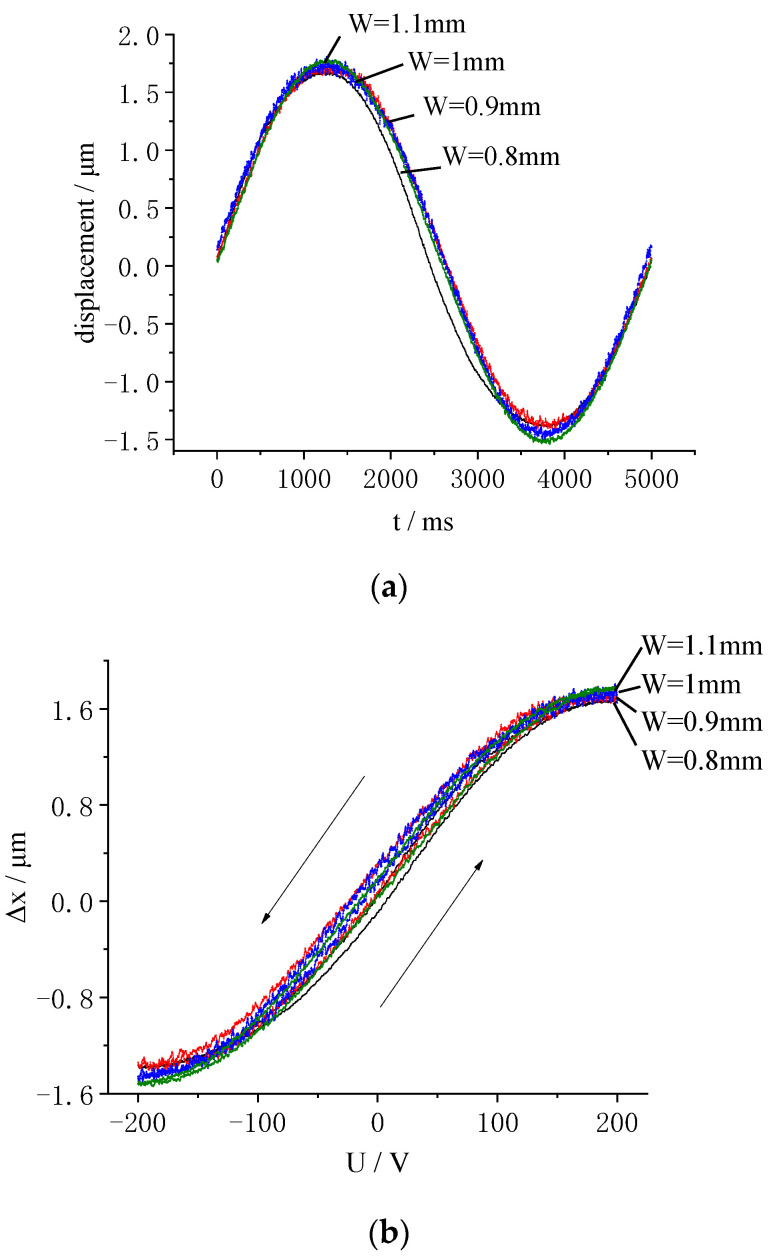
Influence of electrode width on radial displacements: (**a**) Displacement time history versus width; (**b**) Displacement response versus width.

A sinusoidal signal with a frequency of 0.2 Hz and an amplitude of 200 V was applied to the samples. The displacement time history is shown in Figure 14a, and the displacement responses are shown in Figure 14b. As shown in Figure 14b, the middle segment of the curve was linear. As the voltage increased, the displacement increased linearly. There was a high level of repeatability of the curves shown in multiple displacement tests. All the displacement curves were relatively close to each other. As the electrode width increased, the maximum radial elongations of the LRE piezo sample increased from 1.68 μm to 1.81 μm, while the maximum radial contractions increased from 1.39 μm to 1.51 μm. These values did not change significantly with width. Therefore, the influence of the electrode width on the radial displacement of the LRE piezo disk was small. There was a relatively small gap between the rising curve and the falling curve, which was caused by the hysteresis effect of the piezoceramics. During the voltage rise and fall, there was a gap in the displacement curve of the LRE piezo disk. The rising and falling intervals of the displacement response curve did not coincide.

**Figure 15:**
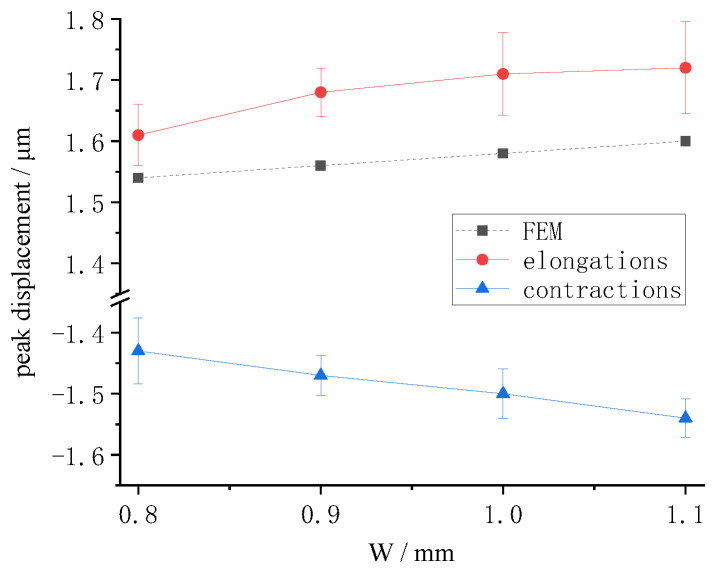
Peak displacements versus the electrode width.

When the voltage was 200 V, the peak displacement was obtained with multiple electrode widths. The finite element results for radial displacement and the test results are summarized in Figure 15. The displacement curve was gentle. The increase in displacement with increase in electrode width was relatively small. Both the test and the finite element analysis were consistent with this law. This was because the electric field increased as the electrode became wider, with the actuator showing better drive performance.

Due to measurement error, the radial displacement under experimental conditions would be larger. The piezoelectric constant used in finite element analysis is relatively small. The assumption of piezoelectric constant consistency causes errors in finite element models. These errors cause finite element analysis results to be small.

#### 4.2.4. Effect of Electrode Center Distance and Number of Rings on Displacement

**Figure 16:**
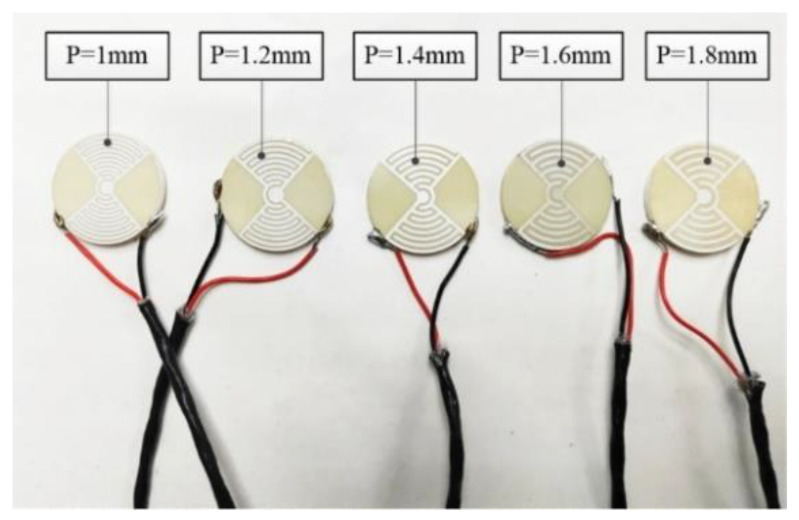
LREs piezo sample with different center distance.

The electrode center distance is another key dimension. It affects the number of electrode rings on the sample surface because the displacement of the LRE piezo disk is superimposed by the displacement of each piezo ring. To research the effect of the electrode center distance and the number of rings on displacement, several samples were prepared. The piezo sample dimensions were Ø25 mm × 2 mm. The width of the electrodes was 0.8 mm, and the electrode center distances were 1 mm, 1.2 mm, 1.4 mm, 1.6 mm and 1.8 mm, in contrast to distances of 11, 9, 8, 7 and 6 for the piezo rings. The samples are shown in Figure 16.

**Figure 17:**
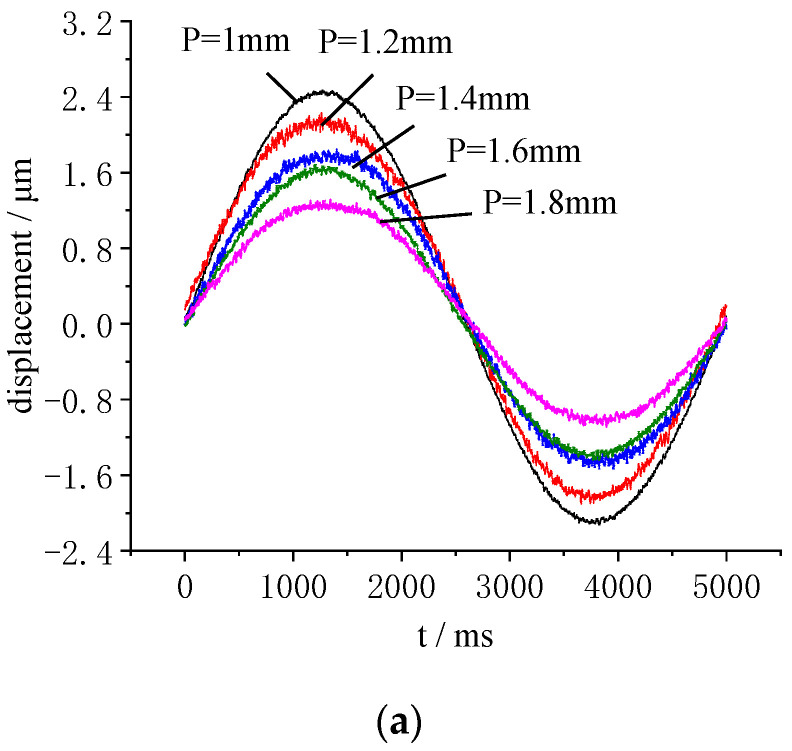

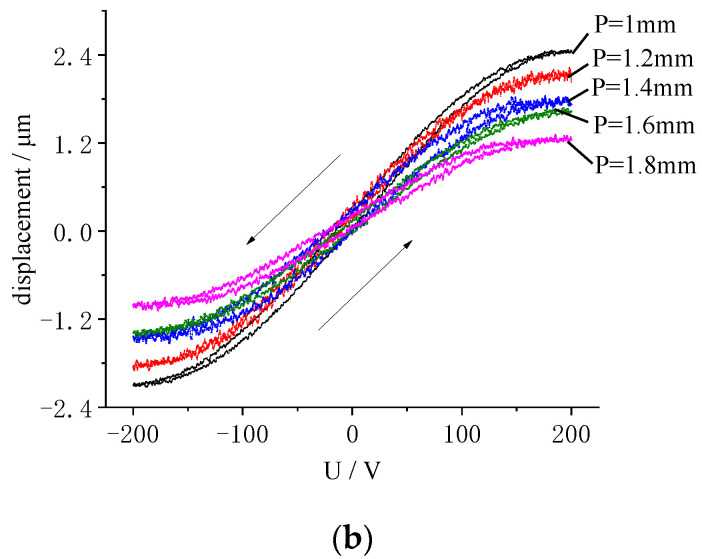
Influence of the electrode center distance on radial displacements: (**a**) Displacement time history versus the electrode center distance; (**b**) Displacement response versus the electrode center distance.

A sinusoidal voltage of 0.2 Hz and 200 V was applied to the samples. The displacement time history and the displacement response are shown in Figure 17. As can be seen from Figure 17, with decrease in electrode center distance, and increase in piezo rings, the radial displacement of the LRE piezo disk was significantly improved. When P was 1.8 mm, the maximum displacement was only 1.32 μm. When P was 1 mm, the maximum displacement reached 2.47 μm. At the same time, the maximum contractions increased from 1.09 μm to 2.13 μm.

**Figure 18:**
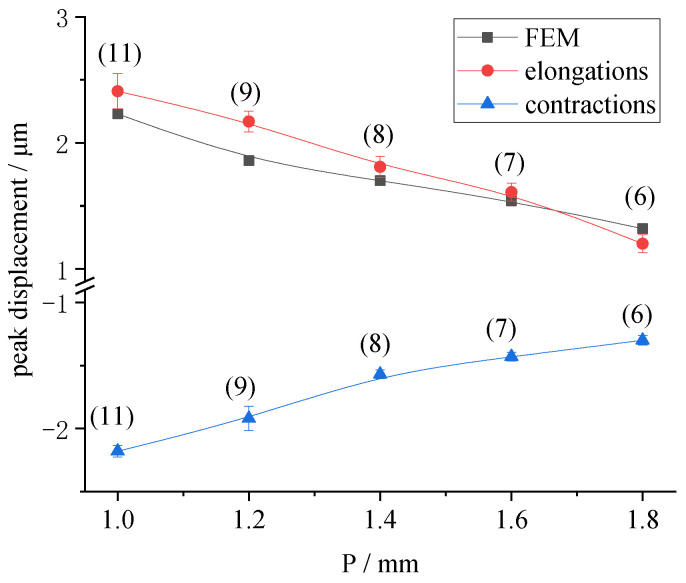
Peak displacements versus the electrode center distance.

The radial displacements of the actuator with the same structure were obtained by static analysis of ANSYS. The finite element results of the displacement were compared with the maximum value of the displacement response obtained in the test, as shown in Figure 18. As the center distance decreased and the number of piezo rings increased, the radial displacement increased significantly. These results jointly confirm the effect of electrode center distance on the radial displacement of LREs piezo disks. The curves are very close, indicating that the finite element model was accurate. Due to error in the preparation of samples and experimental error in displacement detection, as well as the assumptions and calculation accuracy of the finite element analysis, the experimental value of the displacement was also slightly larger than the finite element results.

## 5. Conclusions

To improve the radial displacement of piezo disks, a new piezo actuator with local ring electrodes was proposed by combining interdigitated electrodes with a PZT-52 piezo disk. According to the structural characteristics of the actuator, the factors affecting radial displacement were investigated for the piezo rings and the actuator. The displacement response of the actuator under single electrode excitation and full electrode excitation was obtained. Both finite element analysis and experimental investigation showed that the contribution of the outer piezo ring to the actuator displacement was greater than that of the inner ring. The variation law of radial displacement with radius was found to be basically the same. Under a voltage excitation of 0.2 Hz and 200 V, the peak value of radial displacement for the new actuator was 1.63 μm. This value was 2.5 times that of a conventional electrode piezo disk of the same size. This indicated that the new disk had greater radial displacement. The effect of electrode size on the radial displacement of the actuator was studied by finite element analysis and experiment. The results showed that the influence of electrode width on the driver was small. There was a linear relationship between the center distance of the branch electrodes and the peak value of radial displacement. The experimental results for the influence of the electrode structure on displacement were close to the finite element analysis results, demonstrating that the finite element model of the characteristics of the piezo ring and the actuator has reference value for the design and preparation of LRE piezo disks.

## Figures and Tables

**Table 1:** Dielectric and piezoelectric constants of PZT-52.

Relative Dielectric Constant		Piezoelectric Strain Constant/pC/N		
$\varepsilon_{11}^{T}$	$\varepsilon_{33}^{T}$	*d*_31_	*d*_33_	*d*_15_
3500	3250	260	575	950

**Table 2:** Outer radius of piezo rings.

Piezo Rings	O_1_	O_2_	O_3_	O_4_	O_5_	O_6_	O_7_
r_1/_mm	12.5	10.5	8.9	7.3	5.7	4.1	2.5

## Data Availability

Data are available upon request from the corresponding author.
